# Stem cell therapy for degenerative disc disease: Bridging the gap between preclinical promise and clinical potential

**DOI:** 10.17305/bb.2023.9518

**Published:** 2024-04-01

**Authors:** Matic Munda, Tomaz Velnar

**Affiliations:** 1 Department of Neurosurgery, University Medical Centre Ljubljana, Ljubljana, Slovenia; 2 AMEU-AMC Maribor, Maribor, Slovenia

**Keywords:** Stem cells, degenerative disc disease, spine, intervertebral disc (IVD) regeneration

## Abstract

Stem cell therapy has gained attention in the field of regenerative medicine due to its potential to restore damaged tissue. This article focuses on the application of stem cell therapy for treating spinal pathologies, particularly intervertebral disc (IVD) degeneration. Disc degeneration is a major cause of low back pain and is characterized by changes in the matrix and inflammation. Animal studies have demonstrated that the implantation of mesenchymal stem cells (MSCs) yields promising results, including increased disc height, improved hydration, and reduced inflammation. However, the number of clinical trials remains limited, necessitating further research to optimize MSC therapy. Although preclinical studies offer valuable insights, caution is needed when extrapolating these findings to clinical practice. Stem cell therapy still faces multiple challenges, such as the durability and survival of MSCs upon implantation, uncertain pathways to discogenic differentiation, and the adverse impact of a harsh microenvironment on cell survival. The avascular nature of the IVD and dynamic loading conditions also affect the adaptation of transplanted cells. Despite these obstacles, stem cell therapy holds promise as a potential treatment for disc degeneration, and ongoing research aims to fill the current gap in conclusive data.

## Introduction

In the past decade, stem cell therapy has garnered significant attention in the field of regenerative medicine. The primary objective of stem cell therapy is to replace or restore damaged cells and tissues by facilitating the differentiation of implanted cells into the native cells of the target tissue. These differentiated cells expedite the healing process and contribute to tissue regeneration through self-renewal and paracrine signaling mechanisms [1–4]. The clinical efficacy of hematopoietic stem cell transplantation, already established in the treatment of leukemia and lymphoma, underscores the potential of stem cells in other medical domains as well. Numerous ongoing studies and clinical trials are exploring the feasibility of stem cell therapy as a treatment option for a broad spectrum of neurological disorders. These include neurodegenerative diseases, multiple sclerosis, stroke, intracerebral hemorrhage, traumatic brain injury, spinal cord injuries, and other related conditions [3, 5–7].

**Figure 1.**
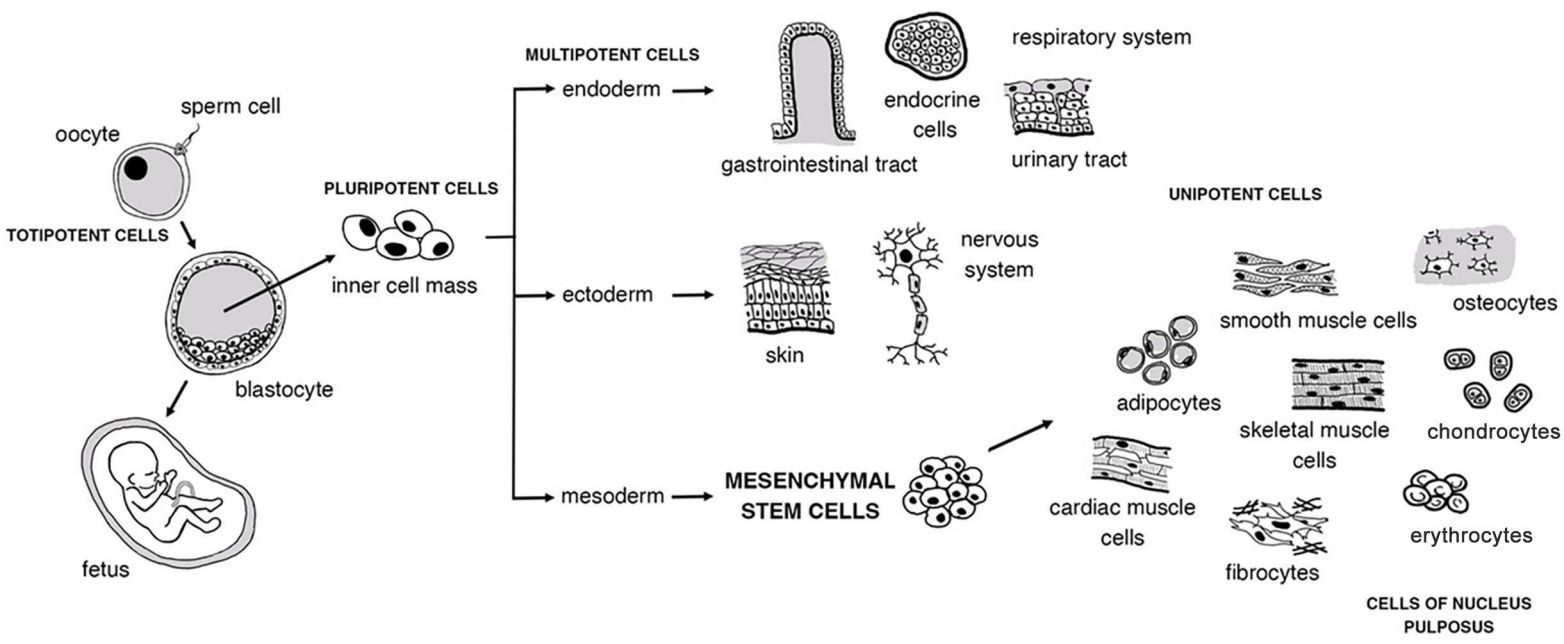
Pathways of cellular differentiation and stem cell lineages.

A wide range of stem cells with varying potentials is available for research purposes. Pluripotent cells, such as embryonic and induced pluripotent cells, have the ability to differentiate into any cell type when exposed to appropriate stimuli. Multipotent cells, including mesenchymal stem cells (MSCs), can differentiate into multiple related cell lineages. In contrast, unipotent cells are limited to differentiating into a single-cell lineage, as is the case with hematopoietic stem cells used for treating leukemia. Embryonic stem cells offer the greatest research potential because of their capacity for self-renewal and differentiation into cell lineages of all three embryonic layers. However, their derivation from the epiblast layer of implanted embryos comes with ethical restrictions that limit their use. Bone marrow-derived MSCs, also known as bone marrow-derived mesenchymal stem cells (BM-MSCs), are most commonly used for research (Figure 1). To obtain BM-MSCs, bone marrow is usually harvested from the posterior dorsum of the pelvic bone, and the aspirate is then centrifuged to concentrate the cells. In addition, MSCs can be sourced from adipose tissue or umbilical cords, and less commonly from synovial, muscle, or periosteal tissues [8–10].

**Figure 2.**
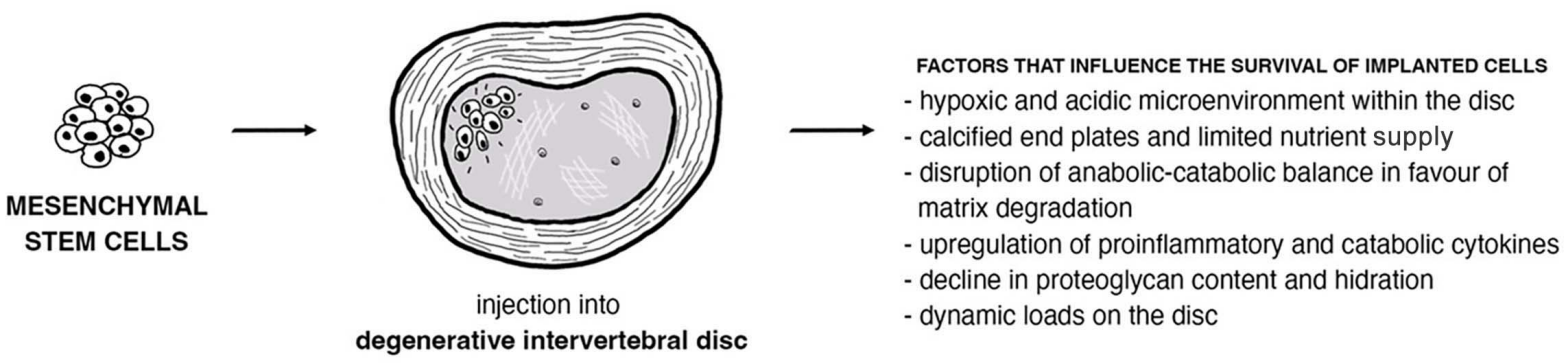
Factors influencing the survival of implanted stem cells.

The major challenge in implanting stem cells into human tissues is ensuring the long-term survival of these cells within the microenvironment, as well as understanding their impact on proliferation and differentiation (Figure 2) [11]. Recent research suggests that the therapeutic effects of implanted stem cells are more influenced by the bioactive factors they secrete than by their direct cellular contributions [12]. Genetic engineering techniques present a promising avenue for enhancing stem cell survival and therapeutic potential. These techniques modify stem cells to express specific growth factors, optimizing their role in tissue reconstruction and prolonging their survival [1, 3]. Various other strategies are also being explored in stem cell implantation research. One area of focus is the development of novel scaffolds or biomaterials that create a favorable environment for stem cells, thereby promoting their survival and guiding their differentiation [13, 14]. Researchers are exploring different delivery methods to improve targeted delivery to specific tissues [15–17]. Moreover, ongoing studies are investigating the possibility of combining stem cell implantation with other therapeutic approaches, such as gene therapy or immunomodulation [4, 18]. Interdisciplinary efforts are underway to advance the field of stem cell implantation and optimize its potential for clinical applications.

Spinal pathology encompasses a variety of conditions, including nerve damage, muscle trauma, disc degeneration, and bone fusion, all of which can significantly impact quality of life. Stem cells, due to their regenerative potential, have emerged as a promising treatment modality for spinal pathologies. The interplay among stem cell survival, differentiation, and their therapeutic impact on spinal conditions requires further investigation. In degenerative cases like disc degeneration, stem cells could be used to attenuate processes that lead to tissue degradation, promote regeneration, and improve functional outcomes without the need for surgical intervention. In spinal cord injuries, stem cell-based approaches show promise in bridging injured areas, facilitating neural regeneration, and enhancing functional outcomes. Although theoretical frameworks and some preclinical studies are promising, the clinical application of stem cells in spinal pathology still lacks substantial evidence. The aim of this article is to highlight the current gaps in conclusive data and emphasize the need for additional research [1, 19–21]. Specifically, we examine factors influencing the long-term survival of implanted stem cells in the spinal microenvironment, elucidate their mechanisms of differentiation, and assess their impact on tissue regeneration. We lay the groundwork for optimizing stem cell use in degenerative disc disorders based on the results of numerous animal and clinical studies.

## Disc degeneration

Disc degeneration begins with a reduction in the population of large notochordal cells within the nucleus pulposus, resulting in altered cellular function within the intervertebral disc (IVD) [22, 23]. This initiates a degenerative cascade characterized by the loss of normal matrix, increased activity of matrix metalloproteinases, a shift from type II to type I collagen, and a decrease in proteoglycan content. Consequently, these morphological changes in the IVD reduce the binding of water molecules to intervertebral components, leading to disc dehydration and subsidence. This contributes to the loss of critical pressures responsible for maintaining the mechanical integrity of the spine, which in turn results in local instability and mechanical trauma. In the course of degeneration, cells in the nucleus pulposus also upregulate the expression of pro-inflammatory and catabolic cytokines, as well as other various inflammatory mediators (e.g., IL-1, IL-6, IL-12, IL-17, TNF-alpha, IFN-beta, and IFN-gamma) [1, 2, 8, 22–26]. These pro-inflammatory cytokines further stimulate the growth of vascularized granulation tissue, cellular remodeling, fibrosis, and the activation of nociceptive nerve endings. Additionally, pathogenesis is further enhanced by other factors, such as ischemia, pressure changes, increased concentrations of catabolic enzymes, and decreased aggrecan levels, among others. The proliferation of nociceptive nerve endings may contribute to the pathological innervation of the disc and the development of discogenic pain [2, 8, 27].

As a consequence, disc depressions, disc herniations, and nerve root compressions occur, further exacerbating pain symptoms [22, 23]. Osteophytes, which form in response to increased pressure loads, contribute to the narrowing of the spinal canal. Lumbar spine pain is the primary symptom of degenerative spine disease, and its treatment usually encompasses pain-relieving therapies, physical therapy, acupuncture, local blocks, lifestyle changes, and, as a last resort, surgery involving IVD removal and spinal fusion [1]. Current treatment options for degenerative disc disease focus on symptom management and providing temporary relief rather than reversing the underlying degenerative process. These approaches include pain medications, physical therapy, steroid injections, and surgical procedures, such as microdiscectomy and artificial disc replacement. In severe cases, laminectomy with or without spinal fusion may be considered. The goal of surgical therapy is to decompress the spinal canal and intervertebral foramen to relieve pain and improve neurological symptoms by releasing the compressed vertebral nerves [1, 2]. Although these methods offer some advantages, they come with operative and postoperative risks, potential complications, and limited success rates. Importantly, these therapeutic options do not address underlying pathophysiological mechanisms, such as inflammation, disturbed matrix balance, and the loss of functional native cells within the IVD. Surgical interventions often result in functional impairments, reduced mobility, and altered spinal biomechanics, which can subsequently lead to new degenerative changes in adjacent discs and the eventual recurrence of pain [2, 23].

As our understanding of the pathophysiology of lumbar spine pain deepens, interest in regenerative medicine is also growing. This field aims to restore natural disc tissue and metabolic balance through cell therapy. Current research focuses on developing biological approaches that directly target the pathogenesis of the disease, with the aim of preventing or treating disc degeneration. Both experimental and clinical trials are underway, investigating the implantation of stem cells into damaged IVDs with the goal of effectively treating LBP [2, 22]. This breakthrough treatment promises not only pain relief but also disc regeneration and the restoration of spinal health. Stem cell therapy represents a paradigm shift in the treatment of degenerative disc disease; stem cells possess the remarkable ability to differentiate into various specialized cell types and regenerate damaged tissue, thus contributing effectively to disc regeneration [22].

Stem cell therapy is considered a promising, revolutionary treatment for degenerative disc disease [23–25]. By harnessing the regenerative potential of stem cells, the hope is to alleviate spinal pain and restore spinal health through some degree of disc regeneration. However, there is still a long way to go before stem cells can be integrated into routine clinical applications for treating degenerative disc disease. Although results from both preclinical and clinical studies to date are encouraging, further research is needed to optimize protocols and demonstrate long-term safety and efficacy. One of the most significant advantages of using stem cells is their unique ability to differentiate into disc-like cells, thus contributing to the regeneration of damaged disc tissue. Therefore, stem cell therapy has the potential to reverse the degenerative process by promoting the growth of new disc cells and restoring the structural integrity of the degenerated disc [24–26]. This regeneration can lead not only to pain relief but also to structural changes in the disc tissue itself, including the restoration of disc height. Anti-inflammatory molecules secreted by stem cells help reduce inflammation and relieve pain associated with degenerative disc disease.

Studies have shown that the positive effects of stem cell therapy for degenerative disc disease can be long-lasting, resulting in sustained pain relief and improved spinal function. However, it is important to note that the duration of symptom improvement remains unknown. The use of autologous stem cells through intradiscal injection eliminates the risk of rejection or disease transmission, making therapy with autologous stem cells a safe and viable treatment option [25, 26]. Stem cell therapy can be performed via a minimally invasive procedure, reducing the risks associated with traditional open surgery. This approach results in shorter recovery times, less postoperative pain, and fewer complications. Stem cell therapy aims to address the root cause of degenerative disc disease, offering the potential for long-term relief [25–27].

## Disc regeneration and animal studies

The cells of the nucleus pulposus within the IVD constitute only 1% of the tissue, yet they play a critical role in producing the extracellular matrix, which is essential for both the morphological integrity of the disc and the biomechanical stability of the spine [22].

The first attempt at autologous reimplantation of nucleus pulposus cells into degenerated IVD was performed in 1998 by Nishimura and Mochida [28] using rats as a model. The implantation of cryopreserved nucleus pulposus cells led to a delay in the degeneration of the annulus fibrosus and preserved the remaining nucleus pulposus compared to the control group [28]. Autologous reimplantation of nucleus pulposus cells has also been shown to slow degenerative changes in additional animal and ex vivo studies, as well as in some human studies. These interventions led to a clinically significant reduction in lower back pain (LBP) and a restoration of disc height and hydration compared to the discectomy-only group [2].

One potential method for treating degenerative disc disease involves the percutaneous implantation of multipotent stem cell therapy. This therapy aims to reconstruct the nucleus pulposus matrix and promote tissue regeneration, thereby relieving nociceptive disc pain and slowing catabolism. MSCs are the most promising cell source for disc regeneration. Numerous studies have shown that both BM-MSCs and adipose-derived MSCs possess the ability to differentiate into a cellular phenotype resembling that of nucleus pulposus cells. These cells function as multipotent immunomodulators and can exert paracrine effects on neighboring native nucleus pulposus cells through the secretion of anabolic growth factors. They also directly produce components of the extracellular matrix, reduce inflammation, and counteract degradation. Bone marrow is the most common source of MSCs, and these cells can differentiate into osteoblasts, adipocytes, or chondroblasts by expressing specific membrane markers in vitro. They can also be differentiated into cells resembling nucleus pulposus cells through genetic modification and stimulation with specific signaling molecules [1, 8, 11, 24]. A 2015 study by Clarke et al. [29] demonstrated that stimulation of BM-MSCs with growth differentiation factor 6 (GDF6) led to significant upregulation of nucleus pulposus marker genes. This facilitated the differentiation of MSCs into cells closely resembling native nucleus pulposus cells [29].

Extensive in vivo studies in animal models have shown that various mechanisms contribute to successful outcomes. For instance, in 2003, Crevensten et al. [30] demonstrated radiographic enlargement of the disc, as well as an increase in cell population and proliferation, by implanting MSCs into artificially degenerated rat discs. Similarly, in rabbit models, Sakai et al. [31] observed the proliferation of injected stem cells and their differentiation into cell types that exhibit the major phenotypic characteristics of nucleus pulposus cells. These differentiated cells synthesized type II collagen and proteoglycans, leading to an increase in disc height and improved hydration [31]. These results underscore the regenerative potential of MSCs to restore disc health.

In addition to cell proliferation and differentiation, MSCs have shown paracrine effects affecting the surrounding native cells of the nucleus pulposus. Studies by Teixeira et al. [32] and Miguélez-Rivera et al. [33] have demonstrated that MSCs can reduce pro-inflammatory cytokines (IL-8, IL-6, and TNF-alpha) through the secretion of bioactive immunomodulatory factors. These paracrine effects play a critical role in creating a favorable microenvironment for disc regeneration [34].

Steffen et al. [35] performed a study using the injection of MSCs into degenerated IVDs in dogs. Although no radiological changes were observed, the treatment led to functional improvements and a reduction in pain. These results suggest that MSCs may have therapeutic effects beyond just structural changes and emphasize the importance of evaluating functional outcomes when assessing the efficacy of such interventions [35].

Overall, both in vitro and in vivo studies in animal models consistently highlight the promising potential of MSC therapy for degenerative disc disease. These studies yield positive results, such as increased disc height, elevated T2 signal on MRI (indicating improved disc hydration), restoration of extracellular matrix content, and reduced inflammation. The studies by Crevensten, Sakai, Teixeira, Miguélez-Rivera, and Steffen collectively contribute to our understanding of the regenerative capabilities of MSCs and underscore their potential as a therapeutic option for degenerative disc disease. It is important to note, however, that the majority of these studies are primarily either preclinical, focusing on pathophysiology and molecular aspects, or based on ex vivo models. While these studies offer valuable insights into the regenerative effects of MSC therapy, caution should be exercised when extrapolating these findings to clinical practice [36].

## Human trials

It should be noted that further research is needed to optimize the use of MSC therapy in the clinical setting. Factors, such as growth factors, anti-inflammatory cytokine antagonists, and intracellular regulatory proteins, have shown promise in in vitro studies but require further investigation for in vivo applications. Clinical trials exploring the potential of MSC therapy in degenerative disc disease have yielded some results (Table 1); however, it is crucial to consider the existing dilemmas that limit their clinical applicability. Despite the positive outcomes, it is important to acknowledge specific concerns regarding its practical implementation [8, 36–38].

**Table 1:** Summary of clinical research exploring the potential of MSC therapy in human trials

**Study**	**Study design**	**Patient population**	**Methods**	**Results**
Yoshikawa et al. [39] 2010	Case reports	Two women aged 70 and 67 years with lumbago and leg pain	Percutaneous injection of autologous BM-MSCs into IVD; Two-years follow-up	Improvement on MRI, higher signal intensity of IVD, alleviation of symptoms
Orozco et al. [40] 2011	Clinical trial	Ten patients with chronic back pain; Average age: 35 years (range 28–42); Four males and six females	Percutaneous injection of expanded autologous BM-MSCs into the nucleus pulposus; One-year follow-up	Improvement of pain and disability (85% of maximum in three months), disc height not recovered radiologically, water content significantly elevated
Pettine et al. [41] 2015	Clinical trial	26 patients; Median age: 40 years (range 18–61); 11 males and 15 females	Autologous bone marrow concentrate disc injection; One-year follow-up	Pain reduction of 33.7% in patients older than 40 years, 69.5% pain reduction of other patients
Centeno et al. [42] 2017	Clinical trial	33 patients with chronic back pain; Median age: 40.3 years (range 19–72); 21 males and 12 females	Injection of culture-expanded, autologous BM-MSCs into IVD; Six-years follow-up	Mean pain improvement of 60% at three years post-treatment, 85% had a reduction in disc bulge size on control MRI
Elabd et al. [43] 2016	Case reports	Five patients with degenerative disc disease; Age range: 25–53 years; Two females and three males	Intradiscal injection of autologous, hypoxic cultured BM-MSCs; Four-to six-years follow-up	Overall improvement in all patients, improvement of strength, 4/5 reported improvement in mobility
Noriega et al. [44] 2017	Randomized-controlled trial	24 patients with chronic back pain randomized into test and control group; Mean age: 38 years; 17 males and seven females	Intradiscal injection of allogeneic BM-MSCs for test group; sham infiltration of paravertebral musculature with anesthetic for control group; One-year follow-up	MSC-treated groups showed quick and significant improvement vs controls, degeneration improved in MSC-treated patients and worsened in controls
Amirdelfan et al. [45] 2020	Multicenter randomized-controlled trial	100 patients with chronic back pain randomized into two test and two control groups in 3:3:2:2 ratio; 53 males and 47 females	Intradiscal injection of allogeneic BM-MSC and BM-MSC with HA for test groups; Intradiscal injection of saline and HA alone for control groups; Three-years follow-up	Significant differences between control and MSC groups regarding pain symptoms and disability index

BM-MSCs: Bone marrow-derived mesenchymal stem cells; IVD: Intervertebral disc; MRI: Magnetic resonance imaging; HA: Hyaluronic acid.

Yoshikawa et al. performed a study with two patients who received autologous BM-MSCs. The patients experienced pain relief and an increase in T2 signal on MRI, indicating signs of disc regeneration [39]. In a study by Orozco et al. with ten subjects, autologous MSCs were implanted into the disc. Although MRI scans did not show significant changes in disc height, there was improved disc hydration and an 85% reduction in pain within the first three months, leading to an overall improvement in quality of life [40]. Similarly, Pettine et al. [41] described statistically significant improvements in pain scores observed in 21 out of 26 patients who underwent autologous MSC therapy. Centeno et al. [42] also reported improvements in pain and a reduction in protrusion on MRIs in a study of 33 patients. Another study by Elabd et al. used preconditioned BM-MSCs cultured in a hypoxic microenvironment after infusing them into the IVD of five patients. All patients reported overall improvement, improvement in strength, and four out of five reported improvement in mobility [43].

Despite the promising reports, there are some notable concerns regarding the studies mentioned. Due to the limited sample sizes and lack of well-designed control groups, it is challenging to make direct comparisons and evaluate the true efficacy of MSC therapy. Additionally, the natural history of degenerative spine disease suggests that spontaneous healing and improvement may occur without any intervention. Without a control group for comparison, it is difficult to attribute the observed results solely to MSC treatment. The studies by Noriega et al. and Amirdelfan et al. attempted to address these limitations by including control or sham groups. Noriega et al. [44] reported improvements in visual analog scale scores (VAS), and MRI analysis showed better outcomes compared to control groups. However, the limited sample sizes and multifactorial nature of the patients in these studies make interpreting the results even more complex. Due to the small number of participants and varying patient characteristics, larger studies are needed to validate these findings.

In addition, a study by Amirdelfan et al. employed a randomized-controlled design and included a larger participant cohort of 100 individuals. The study showed significant differences in pain reduction and overall functional improvement with MSC therapy [45]. However, studies still face numerous limitations and obstacles. These include potential selection bias, unaccounted-for variables, and the need for further long-term evaluations to assess the durability and safety of the treatment. Additionally, the subjective nature of the assessments poses a challenge. Patient-reported outcomes, such as pain perception and functional improvements, are important but subjective and prone to bias. Therefore, it is difficult to draw firm conclusions.

Given these difficulties, while the results of these clinical trials are promising, we emphasize that caution is needed when translating them into routine clinical practice. The lack of control groups, small sample sizes, multifactorial patient conditions, and subjective judgments underscore the need for more rigorous research efforts. Further studies with well-designed control groups, larger sample sizes, objective outcome measures, and long-term follow-up are imperative to provide conclusive evidence and determine the true clinical potential of MSC therapy for degenerative disc disease [37].

**Table 2:** Open questions on stem cell therapy for degenerative disc disease

**Open questions:**
• **Long-Term Survival**: How can we improve long-term survival of stem cells in the avascular disc microenvironment?
• **Mechanisms of Action**: What precise molecular mechanisms underlie stem cell therapy’s therapeutic effects?
• **Differentiation Potential**: Can stem cells differentiation into disc-like cells be optimized or preconditioned for better outcomes?
• **Clinical Efficacy**: What additional clinical evidence is required to establish stem cell therapy’s efficacy?
• **Translational Challenges**: How can the complexities of laboratory success be effectively translated into clinical applications?
• **Optimal Delivery**: What methods ensure accurate stem cell delivery despite disc morphology challenges?
• **Combination Therapies**: How can stem cell therapy be combined with other treatments for enhanced regenerative potential?

**Table 3:** Concluding remarks on stem cell therapy for degenerative disc disease

**Concluding remarks:**
• **Promising Potential**: Stem cell therapy holds promise for treating degenerative disc disorder, offering hope for improved outcomes and reduced chronic pain
• **Preclinical Progress**: Positive results of animal studies and early human trials highlight the regenerative potential of stem cells within the intervertebral disc
• **Clinical Caution**: The gap between preclinical promise and clinical application underscores the need for cautious optimistic and further research
• **Complex Challenges:** Technical limitations, survival issues, and microenvironment complexities demand deeper investigation for effective implementation
• **Revolutionizing Medicine**: Despite challenges, optimized stem cell therapies might have the potential to transform regenerative medicine and disc disease treatment

## Future and dilemmas

The domain of technical limitations and challenges associated with MSC therapy encompasses a range of intricate issues. One such complexity revolves around the efficacy and mechanisms of MSC therapy. Several questions demand exploration: How durable are MSCs after implantation into the disc? Does discogenic differentiation truly occur? What impact does the microenvironment have on their survival and function? Are MSCs directly responsible for tissue regeneration, or do they express bioactive factors that affect other cell functions, as demonstrated in other systems (Table 2)? The IVD represents the largest avascular structure in the human body. With age, as well as endplate degeneration and calcification, the microenvironment of the nucleus pulposus becomes hypoxic and acidic, resulting in limited nutrient supply. Given these conditions and the dynamic loading of the disc, transplanted cells appear to face difficulties in surviving and adapting to the avascular environment within the IVD [2]. The fate of implanted cells within the nucleus pulposus remains unclear. Hang et al. [38] monitored the survival of implanted MSCs in an in vivo canine study using MRI and PET scans, reporting an average survival time of three weeks post-IVD therapy. Additionally, the mechanism by which implanted cells trigger regeneration has not been definitively established. In recent studies, Wang et al. demonstrated in rat models that hypoxic preconditioning affects the survival of implanted MSCs, leading to increases in disc height, collagen type II content, and aggrecan levels. They also observed less apoptosis compared to non-hypoxic preconditioning [46]. These results underscore the importance of preconditioning and optimizing the therapeutic potential of MSCs. By exposing cells to specific environmental factors, such as hypoxia, fluctuations in nutrient availability, or growth factors, MSCs may better adapt to and survive the avascular conditions within the IVD [43, 47, 48].

The origin of human nucleus pulposus cells remains unclear, and the mechanisms for their discogenic differentiation are also largely undetermined. However, numerous studies on nucleus pulposus cell phenotyping have enabled the development of methods for lineage-specific discogenic differentiation of MSCs [29, 49]. Looking ahead, the initial assumption that implanted MSCs would phenotypically differentiate into nucleus pulposus-like cells and replace the target tissue has been challenged by numerous studies. Instead, stem cells have been shown to act primarily in a paracrine manner, through signaling molecules, such as growth factors, cytokines, and extracellular vesicles, exerting their effects on native cells. In particular, exosomes play a crucial role in paracrine signaling; they are involved in various pathological and physiological processes and influence gene expression, migration, proliferation, apoptosis, and receptor cell metabolism. These nanosized vesicles can be released from MSCs and taken up by recipient cells, transmitting their cargo and affecting cell behavior [15–17, 50]. Exhibiting a specific mechanism of action, exosomes serve as carriers of essential cargo from MSCs to recipient cells. This mode of transport plays a significant role in modulating cell behavior and highlights the complex communication that occurs between stem cells and the microenvironment of the IVD. These nuances underscore that the challenges of MSC therapy begin even at the molecular level, emphasizing the ongoing need for further investigation and innovative approaches to fully realize their potential [16, 17, 50]. The development of ex vivo IVD organ models is ongoing. These models provide the advantage of allowing independent control over microenvironmental parameters, thereby facilitating the exploration of the aforementioned challenges. By combining biomaterials and cell therapies with chondrocytes, chondroprogenitor cells, and MSCs, interdisciplinary approaches are being optimistically pursued to achieve breakthroughs in musculoskeletal tissue regeneration. However, translating these models into actual clinical applications necessitates a rigorous validation process, further compounding the complexities of the research [51–53].

Given the increasing prevalence of LBP with age, it is evident that the incidence of IVD-related issues will continue to rise, especially in light of an aging global population, changing lifestyles, and everyday stressors. Therefore, advancing MSC therapy and finding effective solutions for musculoskeletal tissue regeneration, specifically targeting the IVD, is of great importance for the future (Table 3).

## Conclusion

In conclusion, MSC therapies show promise for the treatment of IVD degeneration. However, significant technical limitations exist, including issues related to MSC isolation, expansion, differentiation, and preconditioning. While the clinical and animal studies conducted to date have provided valuable insights into the potential of MSC-based therapies, their scope remains limited. There is an urgent need for additional research to address these existing challenges and expand our understanding of MSC behavior and responses within the complex IVD microenvironment. We emphasize the importance of addressing these limitations in future research. Future investigations should focus on optimizing cell survival, improving functional integration, elucidating mechanisms of action, and refining delivery strategies. Moreover, the development of improved experimental models and translational approaches will be critical to bridging the gap between preclinical studies and clinical applications. Despite current limitations, the enormous potential of MSC therapies and the significant clinical need for musculoskeletal regeneration create ample opportunities for further research and exploration, paving the way for breakthroughs in the field of regenerative medicine.
